# Influencing factors on seroma formation following mastectomy: a retrospective cohort study

**DOI:** 10.1186/s12957-026-04496-z

**Published:** 2026-07-18

**Authors:** Karl Hein, Laura Weydandt, Massimiliano Lia, Senol Dogan, Susanne Briest, Ivonne Nel, Bahriye Aktas

**Affiliations:** 1 Department of Gynecology, Medical Center, Leipzig University, Leipzig, Germany; 2 Comprehensive Cancer Center Central Germany, Partner Site Leipzig, Leipzig, Germany; 3 Department of Medical Data Science, Medical Center, Leipzig University, Leipzig, Germany; 4 Institute for Medical Informatics, Statistics and Epidemiology, Medical Center, Leipzig University, Leipzig, Germany

**Keywords:** Mastectomy, Breast cancer, Seroma formation, SLNB, ALND

## Abstract

**Introduction:**

Seroma is the most common postoperative complication following mastectomy and may result in additional postoperative interventions and increased treatment burden. However, its etiology and predictive factors remain insufficiently understood. This study aimed to identify predictors of postoperative seroma formation.

**Materials and methods:**

We conducted a retrospective analysis of 245 patients (301 breasts) who underwent conventional mastectomy or skin-/nipple-sparing mastectomy, with or without immediate implant reconstruction and axillary surgery, at the University Hospital Leipzig between 2019 and 2023.

Variables analyzed included epidemiological characteristics, neoadjuvant chemotherapy, tumor status, perioperative factors, and wound drainage output. Seroma formation was assessed via drains placed in the breast and axilla. Statistical analyses included univariable and multivariable regression and random forest modeling.

**Results:**

In univariable analyses, higher body mass index (BMI), longer surgical duration, diabetes mellitus, hypertension, advanced tumor stage, and elevated C-reactive protein levels were associated with increased breast seroma formation. Random forest analysis identified BMI, number of resected lymph nodes, surgical duration, hypertension, and diabetes as key predictors, all of which remained significant in multivariable models.

For axillary seroma, BMI, number of resected lymph nodes, and tumor stage were significant in univariable analyses, while BMI and number of resected lymph nodes remained significant in multivariable models.

**Conclusion:**

Seroma formation is primarily influenced by BMI, extent of lymph node removal, and surgical duration, with hypertension and diabetes as additional risk factors for breast seroma.

**Supplementary Information:**

The online version contains supplementary material available at 10.1186/s12957-026-04496-z.

## Introduction

Despite increasing rates of breast-conserving surgery, mastectomy continues to play a crucial role in breast surgery. Important indications are the management of multifocal, inflammatory, and advanced-stage tumors or cases of contraindications to adjuvant radiotherapy following breast-conserving surgery. They are also used as a prophylactic measure, in gender-reassigning surgery or based on an informed patient’s preference [1, 2]. 

A seroma is defined as a collection of serous fluid within unformed tissue cavities [3, 4]. Seroma formation is thought to result primarily from disruption of lymphatic vessels during surgery, combined with inflammatory and transudative processes in the surrounding soft tissue. However, its precise etiology remains incompletely understood [5–7]. Although seromas are the most common postoperative complication after mastectomy [8–10], not all have clinical significance or require an intervention [4]. Nevertheless, clinically significant seromas may lead to numerous secondary complications, including impaired wound healing, delayed recovery, inferior aesthetic outcomes, patient discomfort, flap necrosis, wound dehiscence, increased susceptibility to infection and sepsis, repeated clinical visits, implant revision surgery delays in the initiation of adjuvant therapy and chronic encapsulated seroma [6, 7, 11–13].

Previous studies have investigated a wide range of surgical techniques and perioperative measures aimed at reducing postoperative seroma formation, including immediate reconstruction, vacuum drainage systems, shoulder immobilization, extent of surgical dissection, surgical devices, flap fixation techniques, use of sclerosants, tranexamic acid, fibrin glue and sealants, octreotide, and pressure garments. While some of these approaches have shown promising results, findings remain inconsistent and no universally effective strategy has been established [14–17]. In addition, studies based on real-world data are rare, often have small sample sizes, consider only a limited number of factors, or report low incidences of seroma formation [18–23]. The aim of the present study was to identify clinical factors associated with seroma formation in patients undergoing mastectomy, with or without reconstruction and with or without axillary treatment.

## Materials and methods

In this retrospective study, patients who underwent mastectomy at the certified breast center of the University Hospital Leipzig from 2019 to 2023 were included. Patients were identified through the institutional database by screening all mastectomy procedures performed between 2019 and 2023. Clinical and perioperative data were subsequently extracted from the electronic medical records. No exclusion criteria were applied. Given the retrospective design of the study and the exclusive use of pseudonymized data, informed consent was not required. In accordance with national regulations (§ 34 Abs. 1 Sächsisches Krankenhausgesetz; § 15 Berufsordnung), the requirement for informed consent was waived. The study was reviewed and approved by the Ethics Committee of Leipzig (329/25-ek, 16.09.2025).

Patients underwent conventional mastectomy, skin-sparing mastectomy, or nipple-sparing mastectomy, with or without immediate implant reconstruction. In patients with cancer, the diagnosis was histologically confirmed by core-needle biopsy (CNB) before surgery. All removed tissues were evaluated by the local pathological institute of the University Hospital Leipzig.

When axillary staging was indicated (according to national guidelines [2]), patients received a sentinel lymph node biopsy (SLNB) if the axilla was clinically and sonographically node-negative. Axillary lymph node dissection (ALND) was reserved for selected patients with clinically node-positive disease, residual nodal disease after neoadjuvant therapy, or more extensive sentinel lymph node involvement (≥ 3 positive lymph nodes). All mastectomies were performed using electrocautery, only the primary incision in the skin was done by scalpel. Lymph and blood vessels were sealed with mono- or bipolar electrocoagulation. Each surgery was performed by a senior breast surgeon or a resident assisted by a senior breast surgeon.

The operated breasts were stabilized with a compression bra immediately after surgery. Physiotherapy commenced on the first postoperative day and was continued until discharge. All patients received at least one Redon drain in the breast wound cavity connected to a negative-pressure collection system. Patients undergoing sentinel lymph node biopsy or axillary lymph node dissection additionally received a separate axillary drain. Drain output was recorded separately for breast and axillary drains whenever applicable. Negative pressure was removed on postoperative day seven at the earliest, drains were removed at a flow rate of less than 30 ml in 24 h. The seroma quantity was detected daily from the drainage bottles. Patients were discharged after drainage removal or earlier if drainage ceased prematurely, while those with complications or increased drainage output were discharged later.

CRP levels were routinely measured approximately 24 h after surgery as part of standard postoperative laboratory testing. All patients received antibiotics with cefuroxime during surgery, in case of a penicillin allergy with clindamycin. Antibiotic treatment was continued for patients with breast implant insertions until the drains were removed. Patients additionally received 4,500 IU of Tinzaparin subcutaneously for thrombosis prophylaxis from the first day after their surgery until discharge.

### Data analysis

Statistical analyses were performed using R (v4.4.2). Data wrangling and tabulation were conducted using the tidyverse and gtsummary packages. Skewed variables (e.g., seroma volume) were square root–transformed prior to regression analyses. Because the primary endpoint was seroma volume, a continuous outcome, linear regression rather than logistic regression was used. Univariable and multivariable linear regression models were fitted to estimate beta coefficients with 95% confidence intervals, representing the change in seroma volume per unit increase in each predictor. In addition, random forest analysis was performed to rank predictor importance and account for potential nonlinear relationships between variables. To reduce model complexity and the risk of overfitting, the five highest-ranking variables identified by random forest analysis were selected for inclusion in the multivariable regression models. This approach was used as an exploratory variable-selection strategy rather than to infer causal importance.

The type of axillary surgery (SLNB vs. ALND) was not included in the regression models because it was highly correlated with the number of resected lymph nodes. The latter was chosen as a continuous measure reflecting the extent of axillary surgery and lymphatic disruption.

Random forest models were fitted using the party package (cforest) with 1500–2000 trees and permutation-based variable importance. Interactions were explored using stratified scatterplots and formally tested with interaction terms in linear regression models. All figures were generated using ggplot2. Statistical significance was defined as *p* < 0.05.

## Results

A total of 245 patients (236 women, 9 men) who underwent mastectomy were included in the analysis. Bilateral mastectomy was performed in 56 female patients leading to a total of 301 operated breasts, each of which was analyzed individually in the statistical analysis.

A total of 215 breasts (71.4%) were operated on for invasive breast cancer and 24 (7.9%) because of a carcinoma in situ. While some breasts (*n* = 62; 20.6%) were operated on for risk-reducing or gender-affirming indications and therefore were not associated with breast cancer. Five patients (2.0%) had advanced breast cancer disease; thus, a palliative mastectomy was performed.

The median seroma volume in the axilla was 55 ml (IQR: 0–250) and 455 ml (IQR: 295–660) in the breast. Detailed descriptive statistics for all clinical, pathological, and perioperative variables are provided in Table 1.

**Table 1:** Patient characteristics data are given as median (interquartile range) and *n* (%)

Characteristic	*N* = 301 (%)
age (years)	51 (IQR: 37–69)
BMI (kg/m^2^)	25.4 (IQR: 21.8–29.0)
hypertension	
no	201 (66.8)
yes	100 (33.2)
smoking	
no	243 (80.7)
yes	55 (18.3)
diabetes mellitus	
no	268 (89.0)
yes	33 (11.0)
diagnosis	
invasive breast cancer	215 (71.4)
carcinoma in situ	24 (7.9)
prophylactic mastectomy	62 (20.7)
pT-stage	
0*	109 (36.2)
1	89 (29.6)
2	65 (21.6)
3	30 (10.0)
4	7 (2.3)
pN-stage	
0	132 (43.9)
1	50 (16.6)
2	24 (8.0)
3	6 (2.0)
No axillary surgery	89 (29.6)
number of resected lymph nodes	4.5 (IQR: 2.0–12.0)
CRP-levels 24 h after surgery (mg/L)	13 (IQR: 7–24)
duration of surgery (min)	163 (IQR: 117–216)
breast implant	
yes	156 (51.8)
no	145 (48.2)
Neoadjuvant therapy	
chemotherapy	90 (39.9)
endocrine	84 (27.9)
none	127 (42.2)
total volume of seroma in axilla (ml)	55 (IQR: 0–250)
total volume of seroma in breast (ml)	455 (IQR: 295–660)

Unknown data for: pT-stage: *n* = 1; CRP-levels: *n* = 6; breast implant: *n* = 3

* Prophylactic or gender reassigning mastectomy or after neoadjuvant chemotherapy

### Seroma formation in the breast

In univariable linear regression analyses, several clinical factors were significantly associated with increased breast seroma volume. Specifically, higher BMI (*p* < 0.001), longer surgical duration (*p* < 0.001), presence of diabetes mellitus (*p* = 0.005), hypertension (*p* = 0.009), advanced pT-stage (*p* = 0.017) and pN-stage (*p* = 0.014), as well as elevated postoperative C-reactive protein (CRP) levels (*p* = 0.021), were positively associated with breast seroma volume. The results of the univariable regression analyses are presented in Table 2.

**Fig. 1:**
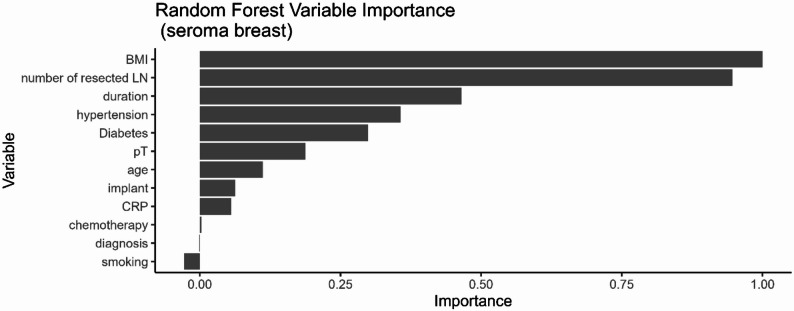
Random forest analysis determining variable importance for breast seroma formation

To further assess variable importance, a random forest model including all clinical predictors was fitted (Fig. 1). The five strongest determinants of breast seroma volume, based on permutation-based importance measures, were BMI, number of resected lymph nodes, duration of surgery, hypertension, and diabetes mellitus.

These five predictors were subsequently included in a multivariable linear regression model. All remained independently associated with breast seroma volume: BMI (*p* < 0.001), number of resected lymph nodes (*p* < 0.001), duration of surgery (*p* < 0.001), hypertension (*p* = 0.007), and diabetes mellitus (*p* = 0.013). An overview of the multivariable results is provided in Table 2.

**Fig. 2:**
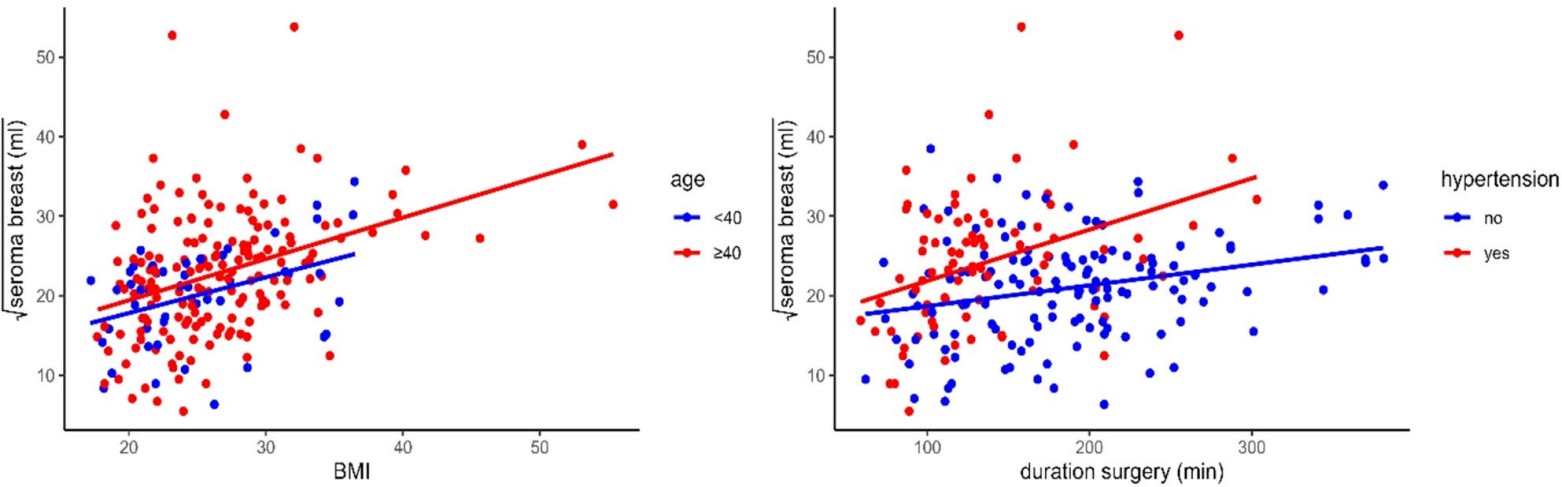
**A** Stratified scatter plot of breast seroma volume by BMI and age group, **B** Stratified scatter plot for breast seroma volume by duration and hypertension

In stratified analyses, the association between BMI and breast seroma volume varied by age group (Fig. 2A). No significant association was observed in patients younger than 40 years (*n* = 94; *p* = 0.228), whereas a significant positive association was found in patients aged ≥ 40 years (*n* = 207; *p* < 0.001). The difference in slopes between age groups was statistically significant (interaction *p* = 0.030), indicating that the association was stronger in older patients.

Similarly, a positive association between surgical duration and breast seroma volume was observed when stratified by hypertension status (Fig. 2B). In both hypertensive and normotensive patients, longer surgical duration was associated with increased seroma volume (both *p* < 0.001); however, the increase in seroma volume per unit increase in surgical duration was significantly greater in hypertensive patients (interaction *p* = 0.007). A summary of regression coefficients and p-values for these stratified analyses is provided in Table S1.

**Table 2:** Univariable regression analysis for seroma in the breast and values of multivariable analysis for the 5 most important parameters due to random forest analysis

Characteristic	Beta (95% CI)	*p*-value	Beta (95% CI)	*p*-value
age (years)	3.7 (0.71 to 6.6)	0.015		
BMI (kg/m2)	24 (16 to 33)	< 0.001	16 (7.8 to 24)	< 0.001
smoking				
no	—			
yes	30 (-106 to 167)	0.66		
diabetes mellitus				
no	—		—	
yes	250 (103 to 396)	< 0.001	184 (39 to 328)	0.013
hypertension				
no	—		—	
yes	178 (74 to 283)	< 0.001	151 (41 to 260)	0.007
diagnosis				
invasive breast cancer	—			
carcinoma in situ	-120 (-300 to 59)	0.19		
prophylactic mastectomy	-139 (-682 to 403)	0.61		
pT-stage				
0*	—			
1	49 (-94 to 192)	0.50		
2	149 (-3.3 to 301)	0.055		
3	249 (67 to 432)	0.008		
4	438 (134 to 742)	0.005		
pN-stage				
0	—			
1	170 (48 to 291)	0.006		
2	254 (92 to 417)	0.002		
3	469 (163 to 774)	0.003		
duration of surgery (min)	0.93 (0.16 to 1.7)	0.018	1.6 (0.90 to 2.4)	< 0.001
breast implant				
yes	—			
no	5.3 (-101 to 112)	0.92		
number of resected lymph nodes (n)	17 (9.1 to 26)	< 0.001	15 (7.6 to 23)	< 0.001
neoadjuvant therapy				
chemotherapy	—			
endocrine	47 (-83 to 177)	0.48		
none	95 (-29 to 220)	0.13		
CRP-levels 24 h after surgery (mg/L)	4.5 (1.1 to 8.0)	0.011		

“Beta” coefficients represent the change in square root–transformed seroma volume per unit increase in the predictor

* prophylactic or gender reassigning mastectomy or after neoadjuvant chemotherapy

### Seroma formation in the axilla

In univariable linear regression analyses (Table S2), higher BMI (*p* < 0.001), advanced pN-stage (all *p* < 0.001 for stages 1–2), advanced pT-stage (*p* = 0.010 for stage 3), and a greater number of resected lymph nodes (*p* < 0.001) were significantly associated with increased axillary seroma volume. Carcinoma in situ was associated with significantly lower seroma volumes (*p* = 0.019).

**Fig. 3:**
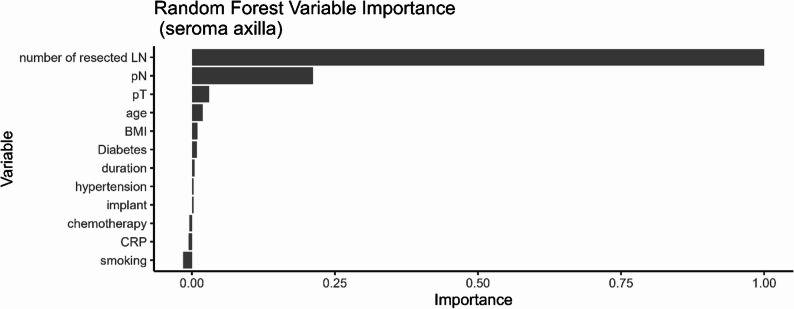
Random forest analysis determining variable importance for axilla seroma formation

The variable importance plot derived from random forest analysis (Fig. 3) indicated that the number of resected lymph nodes and pN-stage were the strongest determinants of axillary seroma volume, followed by pT-stage, diagnosis, and BMI.

These five predictors were subsequently included in a multivariable linear regression model (Table S2). The number of resected lymph nodes (*p* < 0.001) and BMI (*p* = 0.003) remained independently associated with increased axillary seroma volume, whereas diagnosis, pN-stage, and pT-stage were no longer significant.

**Fig. 4:**
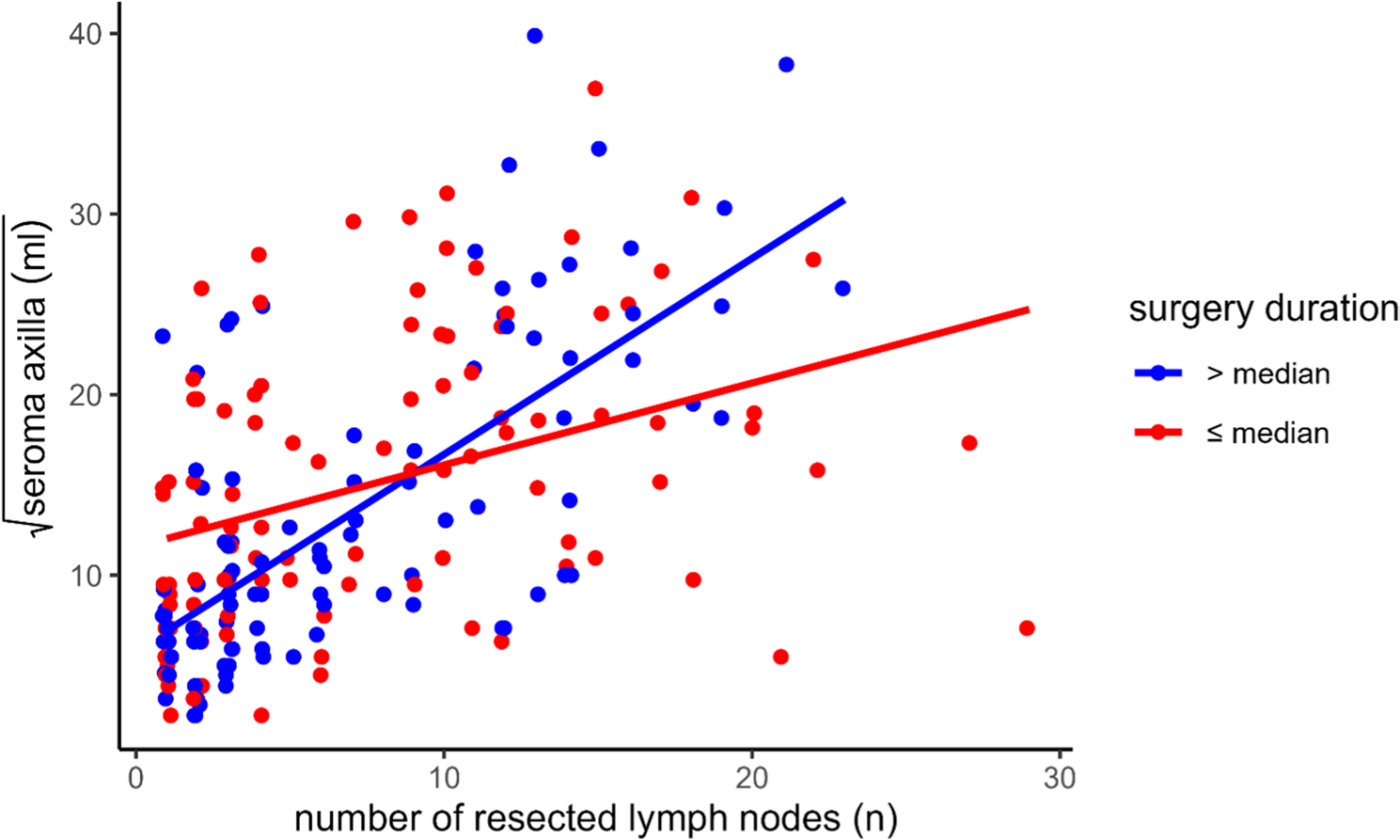
Stratified scatter plot for seroma in axilla, resected lymph nodes by duration, the cut-off was chosen as the median (= 163 min)

In stratified analyses (Fig. 4), the number of resected lymph nodes was positively associated with axillary seroma volume in both subgroups defined by surgical duration. Among patients with shorter operations (≤ median duration, 163 min; *n* = 150), each additional lymph node resected was associated with an average increase of 0.50 ml in seroma volume (*p* < 0.001). This association was stronger in patients with longer surgical durations (> median; *p* < 0.001; *n* = 151). The difference in slopes between groups was statistically significant (interaction *p* < 0.001). A summary of regression coefficients and p-values for these stratified analyses is provided in Table S3.

## Discussion

This study identified key clinical factors associated with seroma formation in both the breast and axilla following mastectomy. For axillary seroma, the number of resected lymph nodes emerged as the strongest determinant in random forest analysis (*p* < 0.001), with increasing lymph node removal associated with higher seroma volumes. This effect was further amplified with longer surgical duration. In addition, higher BMI was independently associated with increased axillary seroma volume (*p* = 0.003).

Regarding breast seroma formation, the most influential factors were a higher number of resected lymph nodes (*p* < 0.001), higher BMI (*p* < 0.001), and longer surgical duration (*p* < 0.001). The association between BMI and breast seroma volume was statistically significant in patients aged ≥ 40 years but not in patients younger than 40 years. Furthermore, interaction analysis suggested that the association was stronger in older patients. Longer surgical duration was associated with increased seroma volume across all patients, with a significantly stronger effect in those with hypertension (*p* < 0.001). Furthermore, the presence of hypertension (*p* = 0.007) and diabetes mellitus (*p* = 0.013) was independently associated with increased breast seroma volume.

The etiology of postoperative seroma formation has been debated for decades. Although some authors consider seroma an unavoidable postoperative consequence rather than a true complication [24], it can result in clinically relevant sequelae, including impaired wound healing, delayed recovery, patient discomfort, flap necrosis, wound dehiscence, increased risk of infection, repeated clinical visits, revision surgery, and delayed initiation of adjuvant therapy [13–16]. According to the Common Terminology Criteria for Adverse Events (CTCAE) version 5.0, seroma is graded from 1 to 3, ranging from asymptomatic collections detectable only by imaging or clinical examination (grade 1) to symptomatic seroma requiring invasive intervention (grade 3) [25].

In contrast to many previous studies, we quantified all postoperative fluid output via surgical drains rather than restricting analyses to clinically symptomatic seroma requiring aspiration (grade ≥ 2), which is common practice in the literature [26, 27]. This approach allowed detection of subclinical (grade 1) seroma, which may be clinically insignificant but remains important for understanding the underlying mechanisms of seroma formation.

Methodologically, we combined traditional regression analyses with random forest modeling to identify the most influential predictors. Random forest models offer advantages over classical regression by reducing overfitting and capturing nonlinear relationships [28]. The five most important variables identified by random forest analysis were subsequently tested in multivariable regression models, confirming their independent associations with seroma volume.

Seroma has been proposed to originate, at least in part, from lymphatic fluid [27, 29]. Our findings are consistent with the hypothesis that lymphatic disruption contributes to postoperative fluid accumulation, as drainage volume increased with the number of resected lymph nodes. However, this interpretation should be made with caution, as several alternative explanations and potential confounders cannot be excluded. Drain output likely reflects a mixture of postoperative fluids rather than pure seroma. Because the biochemical composition of the drained fluid was not analyzed, it remains unclear to what extent the measured volume represents lymphatic fluid, inflammatory exudate, or other postoperative fluid collections. Given the current uncertainty regarding the pathophysiology of seroma formation, our findings should therefore be considered hypothesis-generating rather than definitive evidence for a lymphatic origin of seroma.

The strong association between lymph node removal and axillary seroma underscores the relevance of surgical extent. Axillary lymph node dissection (ALND) inherently involves removal of more lymph nodes than sentinel lymph node biopsy (SLNB), consistent with prior evidence demonstrating higher seroma rates following ALND [30, 31]. Historically, ALND was considered important for oncologic control [32], however, growing evidence suggests that SLNB provides equivalent oncologic safety in many clinical scenarios [33]. Our findings are consistent with previous evidence demonstrating greater postoperative drainage with increasing extent of axillary surgery and reinforce efforts to avoid unnecessary axillary intervention when oncologically appropriate [33–35].

An additional novel finding of this study is the association between postoperative CRP levels and breast seroma volume in univariable analysis (*p* = 0.011). This supports the hypothesis that seroma formation reflects a prolonged inflammatory exudative phase of wound healing [4, 6]. The associations observed for diabetes mellitus and hypertension further reinforce this inflammatory hypothesis. Diabetes is characterized by chronic low-grade inflammation [36], and anti-inflammatory interventions, such as intracavitary methylprednisolone, have been shown to reduce seroma formation [37]. Similarly, hypertension may promote increased exudation through elevated hydrostatic pressure and vasodilation in inflamed tissues [38–40], consistent with our observation that prolonged surgical duration has a stronger effect on seroma formation in hypertensive patients. From a clinical perspective, these findings suggest that optimal perioperative management of diabetes, hypertension, and other inflammatory conditions may help reduce postoperative seroma formation.

Finally, consistent with previous reports [19, 20, 22, 41], higher BMI was associated with increased seroma volume in both the breast and axilla. Notably, the association between BMI and breast seroma was statistically significant in patients aged ≥ 40 years but not in patients younger than 40 years. Furthermore, the interaction analysis suggested that this association was stronger in older patients.Together with the effects of lymph node dissection, surgical duration, hypertension, and diabetes mellitus, these findings suggest that a limited set of clinical variables may help identify patients at increased risk of postoperative fluid accumulation.

Although our study was not designed to evaluate specific drain management strategies, the identified risk factors may have implications for individualized postoperative care. Patients with multiple risk factors, particularly obesity, extensive axillary surgery, prolonged operative times, hypertension, or diabetes, may warrant closer postoperative surveillance and more cautious drain management. Conversely, patients without these characteristics, especially those undergoing less extensive axillary surgery, may represent appropriate candidates for future studies evaluating earlier drain removal or drain omission [16, 42].

In addition, preventive surgical strategies aimed at reducing dead space, such as flap fixation or quilting sutures, may be particularly relevant in high-risk patients [16, 43, 44]. As these techniques were not assessed in the present cohort, no conclusions regarding their effectiveness can be drawn from our data. Future prospective studies should investigate whether risk-adapted approaches to drain management, drain removal thresholds, drain duration, or preventive measures such as quilting can reduce postoperative morbidity. Validation of a predictive model incorporating BMI, extent of lymph node surgery, operative duration, and relevant comorbidities may further support individualized perioperative decision-making.

Several limitations should be considered when interpreting our findings. First, the retrospective design precludes causal inference and relies on the accuracy and completeness of medical records. In addition, definitions and measurement of seroma vary considerably across studies [22–24, 26], limiting direct comparisons with the existing literature.

Second, the absence of exclusion criteria resulted in a heterogeneous cohort, reflecting real-world clinical practice but potentially introducing confounding factors and reducing the precision of subgroup analyses. In particular, patients undergoing immediate implant reconstruction and those undergoing mastectomy without reconstruction represent clinically distinct populations with different indications for surgery, baseline characteristics, and perioperative risk profiles. Although reconstruction status itself was not associated with seroma volume in our analyses, residual confounding related to these differences cannot be excluded.

Third, in patients undergoing concomitant axillary surgery, the breast and axillary wound cavities are often not completely separated and may communicate with each other. As a result, drainage output attributed to the breast or axilla may not represent entirely independent fluid compartments. We therefore evaluated the number of resected lymph nodes as a potential determinant of both breast and axillary drainage volume, as the extent of lymphatic disruption may influence fluid accumulation across the communicating wound cavity. This anatomical overlap may partly explain why the number of resected lymph nodes was associated with drainage volume in both regions and should be considered when interpreting site-specific analyses.

Finally, variable selection for the multivariable models was based on random forest importance rankings. Although this approach enabled identification of the most influential predictors while reducing model complexity, variable importance measures may be affected by correlations among predictors. As a result, some clinically relevant variables may have received lower importance rankings and were not included in the final multivariable models.

Despite these limitations, the combination of conventional regression analyses and machine-learning–based variable selection allowed consistent identification of several clinically plausible predictors of seroma formation. Prospective studies are needed to validate these findings, evaluate their generalizability, and further explore causal relationships.

## Conclusion

We demonstrated that breast seroma formation is primarily associated with higher BMI, a greater number of resected lymph nodes, longer surgical duration, and the presence of hypertension and diabetes. In contrast, axillary seroma formation is mainly driven by the extent of lymph node removal and BMI. Identification of these risk factors may aid improved risk stratification and individualized perioperative management, including tailored drainage strategies and optimization of comorbidities. Prospective studies are needed to validate these findings.

## Supplementary Information

Supplementary Material 1.

## Data Availability

The data presented in this study are available on request from the corresponding author.

## Abbreviations

ALND: Axillary lymph node dissection
BMI: Body mass index
BRCA: Breast cancer gene
CI: Confidence interval
CRP: C–reactive protein
CNB: Core needle biopsy
DCIS: Ductal carcinoma in situ
IQR: Interquartile range
IU: International units
LN: Lymph node
MRM: Modified radical mastectomy
SLNB: Sentinel lymph node biopsy
TNM: Tumor, node, metastasis
