# Supplementary material for: Influencing factors on seroma formation following mastectomy: a retrospective cohort study
Source: World J Surg Oncol. 2026 Jul 18;24:302. doi: 10.1186/s12957-026-04496-z (PMC13380842; doi:10.1186/s12957-026-04496-z)
Supplement: Supplementary file 1 — Supplementary Material 1. [file 12957_2026_4496_MOESM1_ESM.docx]

**Supplementary materials**

| **Analysis** | **Group** | **Beta** | **p-value** | **Interaction p** |
| --- | --- | --- | --- | --- |
| Duration with  hypertension | no hypertension | 0.025 | <0.001 | 0.007 |
|  | hypertension | 0.062 | <0.001 |  |
| BMI with age | <40 years | 0.128 | 0.228 | 0.03 |
|  | ≥40 years | 0.449 | <0.001 |  |

*Table S1: Associations from stratified plots (breast)*

| **Characteristic** | **Beta** **(95% CI)** | **p-value** | **Beta** **(95% CI)** | **p-value** |
| --- | --- | --- | --- | --- |
| age (years) | 1.4 (-0.95 to 3.8) | 0.24 | 0.99 (-1.2 to 3.2) | 0.4 |
| BMI (kg/m2) | 14 (6.8 to 21) | <0.001 | 10 (3.6 to 16) | 0.002 |
| smoking |  |  |  |  |
| no | — |  |  |  |
| yes | 61 (-45 to 168) | 0.26 |  |  |
| diabetes mellitus |  |  |  |  |
| no | — |  |  |  |
| yes | 2.7 (-118 to 123) | 0.97 |  |  |
| hypertension |  |  |  |  |
| no | — |  |  |  |
| yes | 71 (-15 to 157) | 0.10 |  |  |
| pT-stage |  |  |  |  |
| 0* | — |  | — |  |
| 1 | -50 (-166 to 67) | 0.40 | -93 (-200 to 14) | 0.087 |
| 2 | 45 (-78 to 168) | 0.47 | -110 (-236 to 17) | 0.088 |
| 3 | 192 (46 to 339) | 0.010 | -74 (-239 to 92) | 0.4 |
| 4 | -32 (-285 to 222) | 0.81 | -263 (-509 to -18) | 0.036 |
| pN-stage |  |  |  |  |
| 0 | — |  | — |  |
| 1 | 204 (110 to 298) | <0.001 | 112 (-0.45 to 223) | 0.051 |
| 2 | 281 (156 to 406) | <0.001 | 82 (-80 to 243) | 0.3 |
| 3 | 197 (-33 to 428) | 0.093 | -18 (-261 to 224) | 0.9 |
| number of resected lymph nodes (n) | 24 (19 to 30) | <0.001 | 20 (12 to 27) | <0.001 |
| duration of surgery (min) | -0.17 (-0.79 to 0.44) | 0.58 |  |  |
| breast implant |  |  |  |  |
| yes | — |  |  |  |
| no | -66 (-150 to 18) | 0.12 |  |  |
| Neoadjuvant therapy |  |  |  |  |
| chemotherapy | — |  |  |  |
| endocrine | -35 (-138 to 68) | 0.50 |  |  |
| none | -54 (-155 to 46) | 0.29 |  |  |
| CRP-levels 24h after surgery (mg/L) | 2.0 (-0.91 to 4.8) | 0.18 |  |  |

*Table S2: Univariable analysis for seroma in the axilla and values of multivariable analysis for the five most important parameters due to random forest analysis
“Beta” coefficients represent the change in square root–transformed seroma volume per unit increase in the predictor.*

**prophylactic or gender reassigning mastectomy or after neoadjuvant chemotherapy*

| **Analysis** | **Group** | **Beta** | **p-value** | **Interaction p** |
| --- | --- | --- | --- | --- |
| Number of resected lymph nodes with surgical duration | ≤ median duration* | 0.500 | <0.001 | <0.001 |
|  | > median duration | 1.085 | <0.001 |  |

*Table S3: Associations from stratified plots (axilla)
*median duration was 163 minutes*
